# Gravitationally-induced wave function collapse time for molecules

**DOI:** 10.1039/d4cp02364a

**Published:** 2024-07-22

**Authors:** Anderson A. Tomaz, Rafael S. Mattos, Mario Barbatti

**Affiliations:** a Aix Marseille University, CNRS, ICR Marseille France mario.barbatti@univ.amu.fr https://www.barbatti.org; b Institut Universitaire de France Paris 75231 France

## Abstract

The Diósi–Penrose model states that the wave function collapse ending a quantum superposition occurs due to the instability of coexisting gravitational potentials created by distinct geometric conformations of the system in different states. The Heisenberg time-energy principle can be invoked to estimate the collapse time for the energy associated with this instability, the gravitational self-energy. This paper develops atomistic models to calculate the Diósi–Penrose collapse time. It applies them to a range of systems, from small molecules to large biological structures and macroscopic systems. An experiment is suggested to test the Diósi–Penrose hypothesis, and we critically examine the model, highlighting challenges from an atomistic perspective, such as gravitational self-energy saturation and limited extensivity.

## Abstract 2

We compute wave function collapse times based on the Diósi–Penrose hypothesis that gravity induces collapse. These times range from sub-attoseconds in macroscopic bodies to billions of years in isolated molecules.

## Graphical abstract

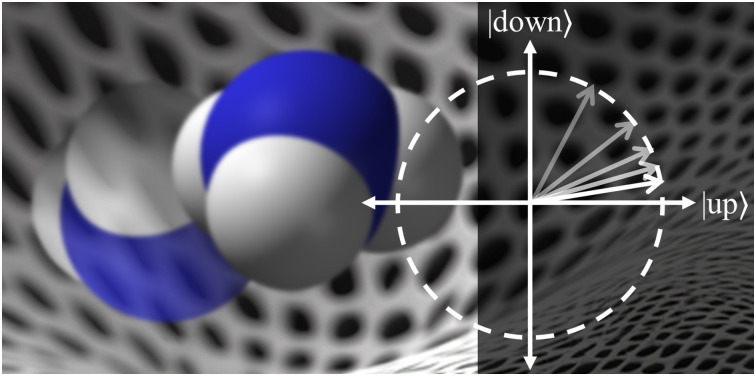

## 1. Introduction

The time-dependent Schrödinger equation and wave function collapse (or reduction) separately determine the time evolution of quantum systems.^1,2^ Since the 1980s, objective collapse theories have become relevant candidates for unifying quantum time evolutions.^3–6^ One of the most intriguing of such theories is the Diósi–Penrose model.^7–10^ Its central hypothesis is that gravity causes the quantum superposition to collapse. When two quantum superposed states are spatially separated, two spacetime distortions associated with each state should co-exist. However, according to Penrose,^9^ such a superposition of distortions is unstable and spontaneously collapses the wave function.

The Diósi–Penrose model holds the highest stakes among the objective collapse theories because it explicitly attributes a physical cause to the wave function collapse, proposing a route to unify quantum mechanics and general relativity.^7–9,11^ If the model is verified, it will profoundly impact fundamental physics. It will not only finally connect the quantum and classical worlds but also explain why all the attempts to quantize gravity have been deceivingly challenging.^9,12^ Indeed, Diósi has recently highlighted exciting parallels between gravitationally-induced collapse and quantum cosmology developments.^13^ For all these reasons, significant effort has been dedicated to understanding the Diósi–Penrose model's theoretical basis and seeking its experimental verification.^14–16^

Our understanding of the transition between quantum and classical worlds is incomplete. Taken separately, quantum mechanics and classical mechanics are astounding successes in their respective validity domains. Nevertheless, ask most physicists how a collection of quantum objects may engender a classical system. We will inevitably listen to a historical digression with terms like “Copenhagen interpretation” and “Many-worlds hypothesis,” followed by a candid admission that we still do not know the answer to the measurement problem or why quantum mechanics describe superpositions, but we only observe definite results.

However, we can do better than that.

A good deal of the quantum-to-classical transition is explained by decoherence.^17^ Decoherence is the delocalization of the coherence of a subsystem over the environment entangled with it. It plays two roles: it selects the preferred basis through environment-induced superselection (*einselection*), which can eventually be measured, and it suppresses any superposition of states on this basis.

**Fig. 1:**
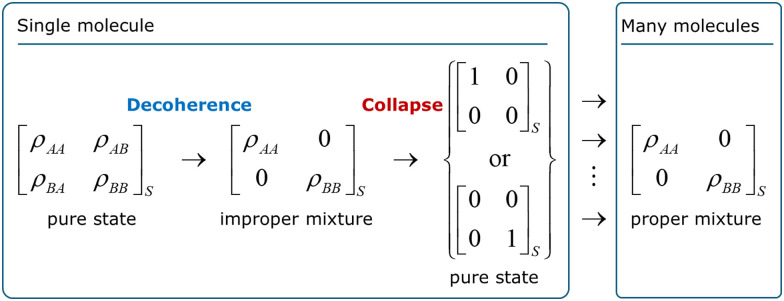
Decoherence and collapse in a two-state molecule. *ρ*_*ij*_ are the elements of the reduced density matrix of the molecule traced over the environment (which is indicated by the subindex S). The environment may be other molecules, electromagnetic fields, or even black body radiation the molecule emits if it is vibrationally excited.

For a molecule, decoherence will occur because of entanglement with other molecules, thermal photons, background radiation, or, if the molecule is vibrationally excited, blackbody radiation it emits.^18^ Decoherence successfully explains why superpositions are not easily observed in large systems. It is formally expressed by the tendency of the nondiagonal terms of the subsystem's reduced density matrix to become zero with time.^19^ For a two-state subsystem, decoherence looks like the process illustrated on the left side of Fig. 1. (Decoherence can also be formulated without referring to reduced density matrices in the frame of environment-assisted invariance (*envariance*).^20^)

After decoherence, only states A or B can be observed, not any superposition of them. Nevertheless, the quantum-to-classical transition is not complete yet in standard quantum mechanics. Decoherence leaves the subsystem in an improper mixture, which still implies that the subsystem may be in either state while we only actually observe one of the states. Thus, one more step is needed to complete the transition, which is the collapse. For the two-state example in Fig. 1, the collapse would definitely bring the system to either state A or B. If one considers an ensemble of identically prepared subsystems, it yields a proper mixture. Although the figure is restricted to the system (as the index S indicates), collapse extends over the entire density matrix, including apparatus, environment, observer, and, in the limit, the universe.

This distinction between decoherence and collapse is not yet fully established in the literature. Sometimes, the terms are used interchangeably^21^ (which is not recommended); in others, decoherence time is taken as a proxy for collapse time^22^ (which is not guaranteed). In this work, we assume that decoherence and collapse are separate processes that may interfere with each other and occur within much different time scales in principle.

At this point, there is no agreement on whether collapse actually exists or if it is even needed. Relative state interpretations^23,24^ (aka many-worlds interpretations) do not require collapse. It assumes that a single quantum world following the unitary Schrödinger evolution exists. However, this quantum world is split into infinite noninteracting branches. The collapse is, thus, an illusion of an observer whose wave function is trapped in one of these branches. Decoherence is a fundamental piece of relative state interpretations,^20^ as it establishes the preferred basis for the branching. Epistemic interpretations also do not require collapse.^25,26^ QBism, for example, considers collapse “nothing but the updating of an agent's state assignment on the basis of her experience.”^27^

The objective collapse theories^3–5^ share the core hypothesis that a wave function is a real object (rather than a way of interpreting reality^28^) and that a quantum superposition of states occasionally collapses into a single state. The unified unitary wave function plus collapse time evolution is achieved by including nonlinear terms in the Schrödinger equation.^4^ These nonlinear terms give rise to a collapse time inversely proportional to the system size, explaining why macroscopic objects are never observed in quantum superposition.

Thus, objective collapse theories are not a mere interpretation of quantum mechanics. They are new theories with standard quantum mechanics as a limit. Moreover, objective collapse is a nonunitary, irreversible process, making it entirely distinguishable from decoherence, which is unitary and (in principle) reversible.

Despite the large body of literature published about the Diósi–Penrose model (the seminal paper by Diósi^29^ has been cited nearly 600 times, and the one by Penrose^8^ received over 1600 citations), the modeling of realistic atomistic and molecular systems is underdeveloped.^15,30^ This paper profits from our experience developing nonadiabatic molecular dynamics methods^31^ to derive general atomistic equations to compute the Diósi–Penrose collapse time. We employ these equations for systems ranging from small molecules to macrostructures, propose an experiment, and critically discuss the Diósi–Penrose model from the molecular perspective. (Note: during the revision of our work, Figurato *et al.*^32^ deposited a preprint with an atomistic approach close to ours, although less general.)

## 2. Diósi–Penrose collapse time

**Fig. 2:**
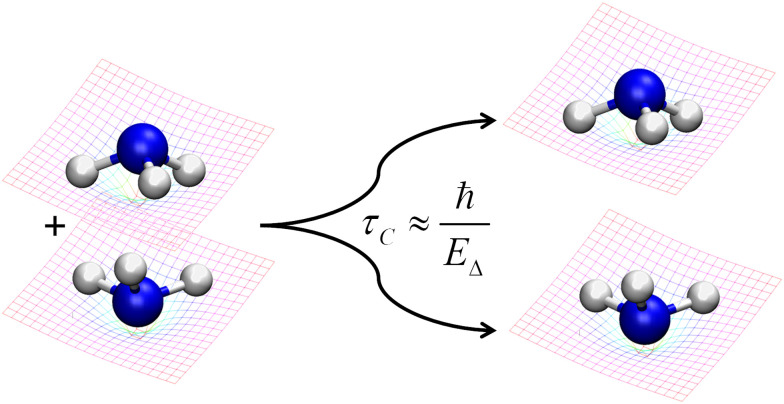
The Diósi–Penrose model predicts that superposed spacetimes, occurring when quantum superposition diverges spatially, are unstable, causing a wave function collapse within *ħ*/*E*_*Δ*_, where *E*_*Δ*_ is the gravitational self-energy of the difference between the two mass distributions. The figure schematically illustrates a coherent superposition of the up and down states of NH_3_ (discussed in Section 3.1), which stochastically collapses to one of the conformations after a time *τ*_C_.

The Diósi–Penrose model's central assumption is that gravity causes the quantum superposition to collapse.^7–10^ If an object is in a superposition of two quantum states with different spatial positions, it implies a gravitational instability, which spontaneously collapses the wave function, with a time constant inversely proportional to the gravitational self-energy of the difference between the mass distributions of the two superposed states (Fig. 2).^8^

Suppose we have a system in a state |ψ〉 that is a superposition between stationary states |A〉 and |B〉^33^

1 |ψ〉 = *c*_A_|A〉 + *c*_B_|B〉

where *c*_A_ and *c*_B_ are complex coefficients (not both zero). Let **a**_A_(**r**) and **a**_B_(**r**) be the free-fall accelerations at point **r** in the respective spacetime of A and B. Penrose heuristically proposed that the energy associated with the difference of these accelerations^8^2
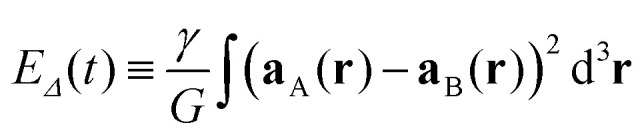
is a measure of the incompatibility between these spacetimes. In this equation, *G* is Newton's gravitational constant and *γ* is a positive, open constant.

For small masses, we can develop *E*_*Δ*_ in terms of the Newtonian gravitational potentials of A and B to obtain (see Appendix A)^16^3
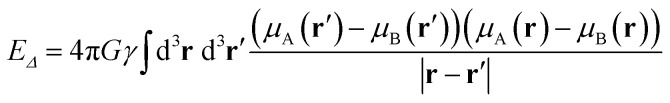
which is the gravitational self-energy of the difference between the mass density distributions *μ*_A_(**r**) and *μ*_B_(**r**) of the two states. Then, following the Heisenberg time-energy uncertainty principle, the Diósi–Penrose collapse time is^7^4
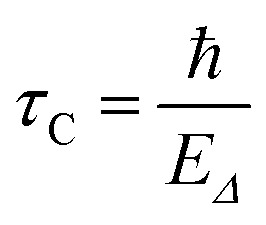


The gravitationally-induced collapse time expressed in (4) has also been derived in other ways. The original proposal is due to Diósi, who gets it from a master equation with gravitational damping term.^29^ It also arises from a conflict between quantum superposition and the equivalence principle of general relativity when one considers the wave function phases of systems evolving in accelerated and free-fall frames.^16^ The collapse time in (4) is strictly valid for stationary states.^9^ Tackling the dynamical evolution of the collapse would require solving the Diósi master equation.

For an ensemble of *N*_at_ nuclei, we can integrate analytically (3) and get a simple expression for *E*_*Δ*_. To do so, we suppose that each nucleus *i* has a Gaussian mass distribution5
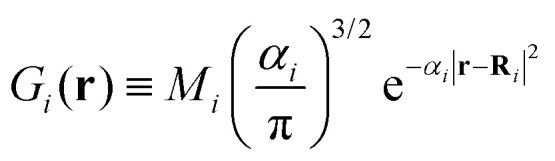
where *M*_*i*_, **R**_*i*_, and *α*_*i*_ are the nucleus mass, expected position value, and Gaussian width. This is an approximation commonly employed in quantum molecular dynamics simulations^34^ to describe the nuclear wave function of a molecule. Note that, despite the formal similarity, the Gaussian length *α*_*i*_^−1/2^ should not be mistaken for the correlation length *r*_C_ discussed in the continuous spontaneous localization model (CSL) literature (see, for instance, ref. 35) and experimentally estimated.^36^

We show in Appendix B that using (5) and neglecting the electron masses, *E*_*Δ*_ becomes6
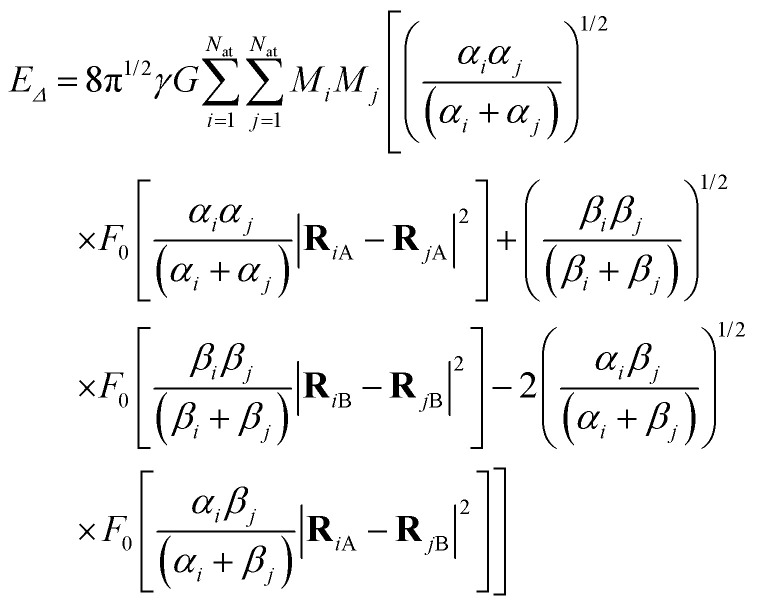
where **R**_*i*A_ and *α*_*i*_ are the expected position and Gaussian width for particle *i* in state A. Equivalently, **R**_*i*B_, and *β*_*i*_ are these quantities for state B. In (6),7
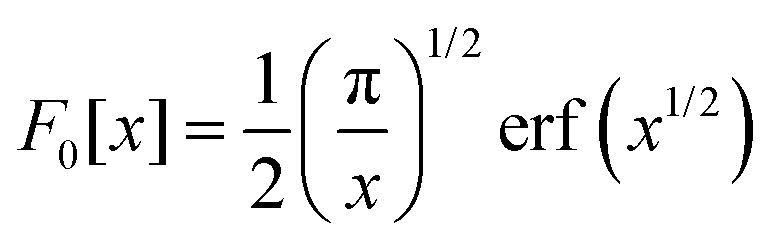


We also show in Appendix C that for a homogeneous medium of identical nuclei of mass *M* and Gaussian width *α*, the gravitational self-energy is8
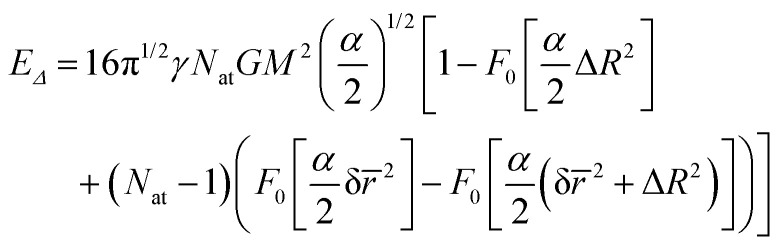
where Δ*R* is the displacement between equivalent atoms in states A and B and δ*r̄* is the mean distance between any pair of atoms. If Δ*R* ≪ δ*r̄*,9
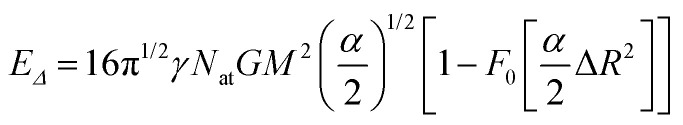


Eqn (6)–(9) are our main theoretical results, derived for the first time here. In the next section, we use them to evaluate the Diósi–Penrose collapse times in different systems.

## 3. Results

### 3.1 Estimating the collapse time

As a first example, let us discuss the symmetry breaking in the isolated ammonia molecule, NH_3_. For each rotational and translational state, the nitrogen atom can be above or below the plan formed by the hydrogen atoms (see Fig. 2). The lowest energy state of a single isolated ammonia molecule |ψ_+_〉 is a symmetric superposition of these two pyramidal states^37,38^10
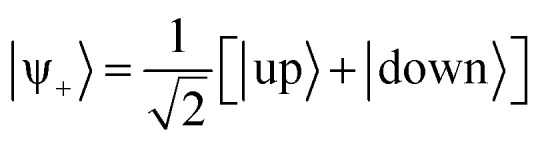


We can estimate the Diósi–Penrose time of this system by directly evaluating its gravitational self-energy with (6). To do so, we need the mass distribution of states |up〉 and |down〉. To get them, we first optimized NH_3_ up-pyramidal ground state geometry. (Here, we did it with density functional theory using the B3LYP functional^39^ and the 6-311G(d,p) basis set.^40^ For a more advanced discussion of NH_3_ geometry, see ref. 41.) The down-pyramidal geometry is obtained by a simple symmetry transformation. Additionally, we must translate both to ensure that their center of mass coincides.

**Fig. 3:**
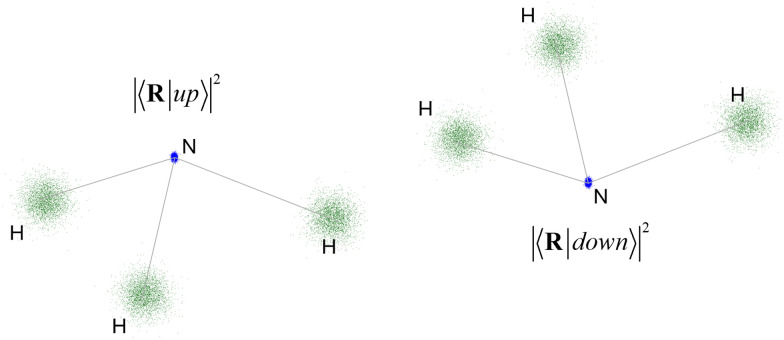
Nuclear density distributions of the up and down pyramidal structures of NH_3_. The straight lines are to guide the eyes only.

Next, we should determine the nuclear mass distributions for these NH_3_ structures. We follow the approach Penrose proposed,^9^ where the nuclear wave function determines the mass distribution. The experiments of Donadi *et al.* have contested this approach,^15^ and we return to this point later in Section 3.3. Thus, each nucleus is represented by a Gaussian function with the widths given in Table 1. These distributions are illustrated in Fig. 3. For the nitrogen nucleus, with a Gaussian width *α* of 19 *a*_0_^−2^,^42^ the Gaussian standard deviation *R*_*N*_ = (2*α*)^−1/2^ is 0.09 Å. In the case of the hydrogen nucleus (proton), it is 0.17 Å. However, we should be careful when interpreting this result.^43^ It does not mean that the nuclei have this actual size. If they are measured, they collapse into their standard femtometer scales. A better way to interpret the distribution plotted in Fig. 3 is to consider that each dot forming the nitrogen cloud corresponds to a single set of dots in each of the three hydrogen clouds. We cannot pick the dots from each nuclear cloud independently.

**Table 1:** Frozen Gaussian widths *α*, as given in ref. 42–46. The length *R*_*N*_ = (2*α*)^−1/2^ corresponds to the Gaussian standard deviation. For elements not given in the table, *R*_*N*_ = 6255 exp(−*R*_at_/0.139) + 0.0744, where *R*_at_ is the atomic radius (in Å) given in ref. 47. This fitting was obtained by excluding Cl from the dataset

Element	*α* (*a*_0_^−2^)	*R*_*N*_ (Å)	Ref.
H	4.7	0.17	42
C	22.7	0.08	42
N	19.0	0.09	42
O	12.2	0.11	42
F	8.5	0.13	42
Si	16.7	0.07	44
S	16.7	0.09	42
Cl	7.4	0.14	42
Ge	30.0	0.07	45
Br	36.7	0.06	46
Cs	21.0	0.08	46
Pb	24.6	0.08	46

Now that we have a Gaussian representation of the nuclei, the application of (6) is straightforward. The gravitational self-energy of NH_3_ quantum superposition is *E*_*Δ*_ = 1.1 × 10^−51^ J, with *γ* = 1/(8π) as proposed in ref. 16 (see also discussion in Section 3.3). With (4), this extraordinarily tiny energy uncertainty implies an immensely long collapse time of 10^17^ s or 10^9^ years. (This and other collapse times estimated in this work are collected in Table 2.) For comparison, the decoherence time of isolated NH_3_ in perfect vacuum due to thermal photons at room temperature is about 10^20^ s. Thus, under such conditions, the collapse would occur much before the molecule could decohere. (For this estimate, we supposed that the NH_3_ molecule is a small dielectric sphere of radius 1.5 Å, and followed the treatment of Joos and Zeh.^48^) On the other hand, supposing that the best laboratory vacuum has a number density 10^−17^ smaller than standard room pressure,^17^ decoherence toward the residual gas would happen within 10^6^ s, killing any sign of quantum superposition much before the wave function collapsed. (In these decoherence estimates, we neglected the recoil of the molecule caused by the environmental scattering. This is a valid approximation for photons but not suitable for a gas. Still, the difference in many orders of magnitude between collapse and decoherence times in the residual gas should be observed if recoil were considered.)

**Table 2:** Some of the Diósi–Penrose collapse times estimated in this work

System	Number of atoms	Collapse time (s)
NH_3_ isomerization	4	1.0 × 10^17^
C_20_ diffraction	20	1.3 × 10^15^
C_720_ diffraction	720	8.4 × 10^12^
Neuron activation	10^6^	1.1 × 10^10^
Iron vacancies	1.8 × 10^15^	1
Aluminum pointer	2 × 10^22^	6 × 10^−21^

Let us now consider the quantum superposition that emerges when a medium-sized molecule like C_70_ diffracts as a matter wave.^49^ These impressive experiments by Arndt, Zeilinger, and co-workers detected such waves using a Talbot–Lau interferometer with gold gratings with a 991 nm period of 476 nm slits. The successful detection of interference fringes with about 40% visibility implies that C_70_ was in a state superposition involving at least two neighbor slits. More recently, Arndt and colleagues used a similar setup to demonstrate quantum interference for molecules with up to 2000 atoms.^36^

Using the fullerene geometries from ref. 50 (available at nanotube.msu.edu/fullerene), we estimated the Diósi–Penrose collapse time of diverse fullerenes under conditions like those of the Talbot–Lau interferometer. We only considered interference between two neighbor slits of the grating, simplifying the estimate to a two-state problem. The original fullerene geometry was assumed to correspond to one of the states. For the second state geometry, we rigidly translated the original geometry along direction *x* by 991 nm. This information and the Gaussian width for carbon nuclei (given in Table 1) are all we need to compute the gravitational self-energy with (6) and the collapse time with (4).

**Fig. 4:**
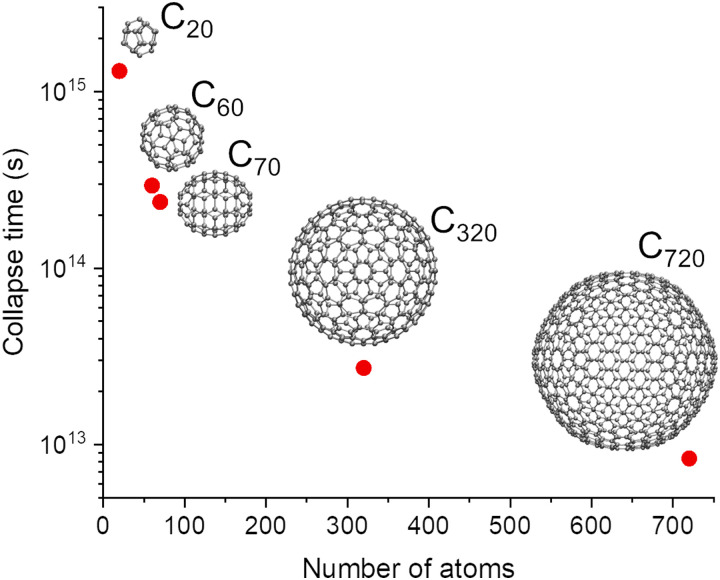
Diósi–Penrose collapse times for diverse fullerenes crossing a grating with slits separated by 991 nm.

Fig. 4 shows the Diósi–Penrose collapse times for diverse fullerenes between C_20_ and C_720_. The collapse time for C_70_ is 2.4 × 10^14^ s. If the experiment were repeated with C_720_, a much larger buckyball, the collapse time would be 8.4 × 10^12^ s.

**Fig. 5:**
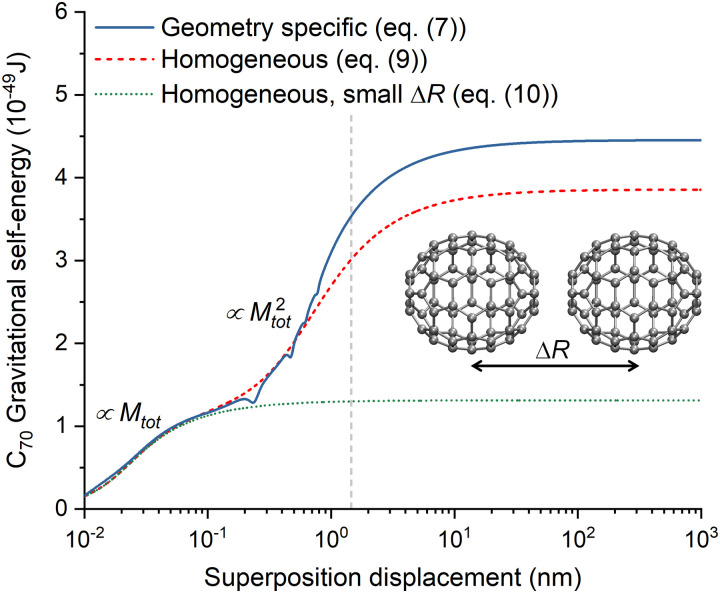
Gravitational self-energy difference for C_70_ as a function of the superposition displacement Δ*R*. The vertical line indicates the value at which the fullerene in state A touches that in B in a single point.

Before moving to another application, let us check the effect of the approximations introduced in the homogeneous models (8) and (9) compared to the geometry-specific model (6). Fig. 5 shows *E*_*Δ*_ computed with the three models as a function of the superposition displacement Δ*R* for C_70_ displaced along the *x* direction in the coherent states. The homogeneous model (8) was computed with the mean interatomic distance of C_70_, δ*r̄* = 0.503 nm. It is in agreement with the geometry-specific model, showing similar self-energy saturation values (see also discussion in 3.3). At the maximum *E*_*Δ*_, the collapse times of the two models are 2.4 × 10^14^ (geometry specific) and 2.7 × 10^14^ s (homogeneous). The homogeneous model in the approximation δ*r̄* ≫ Δ*R* (eqn (9)) agrees well with the other two results for small values of Δ*R* but diverges later, with a much lower saturation *E*_*Δ*_, corresponding to a collapse time of 8.0 × 10^14^ s.

At small Δ*R* values, the *E*_*Δ*_ in Fig. 5 grows proportional to the total mass *M*_tot_. In contrast, for larger values, it does with *M*_tot_^2^. The reason for this difference is that for small Δ*R* values, the (*N*_at_ − 1)^2^ crossing terms involving states A and B in (6) tend to cancel out, leaving only the *N*_at_ same-atom terms. Indeed, this feature motivates deriving (9) from (8).

Consider now quantum superpositions in a neuron. The active (firing) and inactive (resting) states of this type of cell are distinct by the sodium ions that can be inside the axon in the active or outside in the inactive state.^51^ The inside/outside positions Δ*R* are separated by an ion channel membrane (the nodes of Ranvier) 8 nm wide. The number of Na^+^ ions involved in the activation is about 10^6^, distributed over all nodes, each one with about 1 μm length (which we take as 2δ*r̄*). Thus, supposing that we have a coherent state composed of the active/inactive state superposition, where only the sodium ions contribute to the gravitational self-energy, we can employ (9) to estimate the collapse time. Using the fitting proposed in Table 1, *α* = 24.9 *a*_0_^−2^ for the atomic radius *R*_at_ = 2.25 Å,^47^ yielding a collapse time of about 10^10^ s. This value is tremendously longer than the axon recovery time (10^−3^ s) and decoherence time due to water molecules and other ions in the environment, estimated in 10^−20^ seconds by Tegmark.^51^ This decoherence estimate should be taken cautiously as it does not consider collective effects specific to the molecular structure. For instance, Tegmark also used a similar approach to estimate the decoherence in microtubules in 10^−13^ s.^51^ However, a recent study by Babcock *et al.* detected superradiance effects in microtubules due to the collective interactions protecting the network against decoherence for more extended periods.^52^

**Fig. 6:**
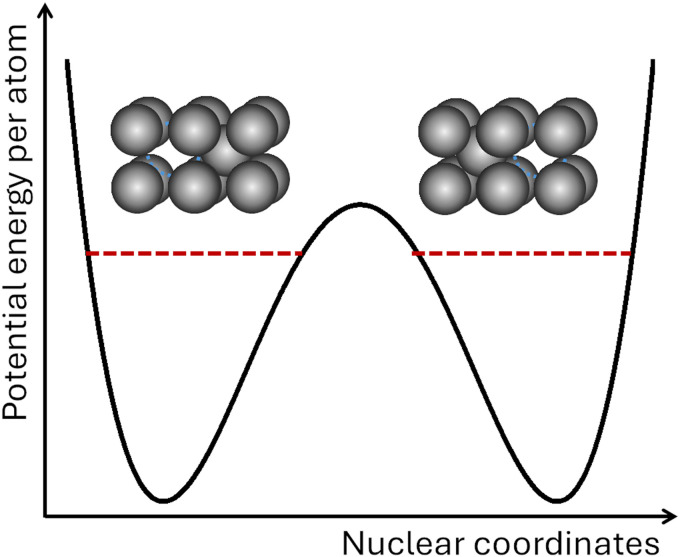
Schematic double-well potential separating bcc iron structures with vacancies at left and right. The dashed circle indicates the vacancy.

As another example, let us gauge how long it takes for the wave function of a large chunk of matter to collapse, according to the Diósi–Penrose model. Consider a piece of iron crystal in its lowest-energy bcc structure (α-Fe) with vacancies,^53^ as illustrated in Fig. 6. In principle, these two iron structures can exist in a quantum superposition of the type |ψ〉 = *c*_1_|left〉 + *c*_2_|right〉.

We will not mind the geometric details of the structures and suppose that the atom involved in the defect is displaced by 2.8 Å between the two states, and all the remaining atoms remain fixed. Given a vacancy concentration of 10^14^ mol^−1^ and a crystal volume much bigger than the superposition displacement, we can use (9) for the homogeneous medium to estimate the Diósi–Penrose collapse time as a function of the number of atoms (and, equivalently, the total mass).

We must, however, note that not all atoms in the medium are displaced by the superposition; only those creating the left/right states are displaced. Thus, we split the iron atoms into two sets: those in the system (which includes the *N*_S_ = 10^14^ atoms per mol that have different positions in the superposition) and those in the environment (all remaining atoms that remain in the same position). With such a split, the difference in mass distribution that appears in (3) is11
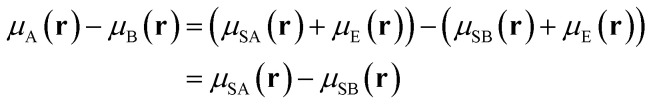
where S and E stand for system and environment. Eqn (11) implies that only the system's gravitational self-energy contributes to the Diósi–Penrose collapse time. Therefore, we should replace *N*_at_ with *N*_S_ in (8). Using the fitting proposed in Table 1, *α* = 24.9 *a*_0_^−2^ for the atomic radius *R*_at_ = 2.26 Å.^47^

**Fig. 7:**
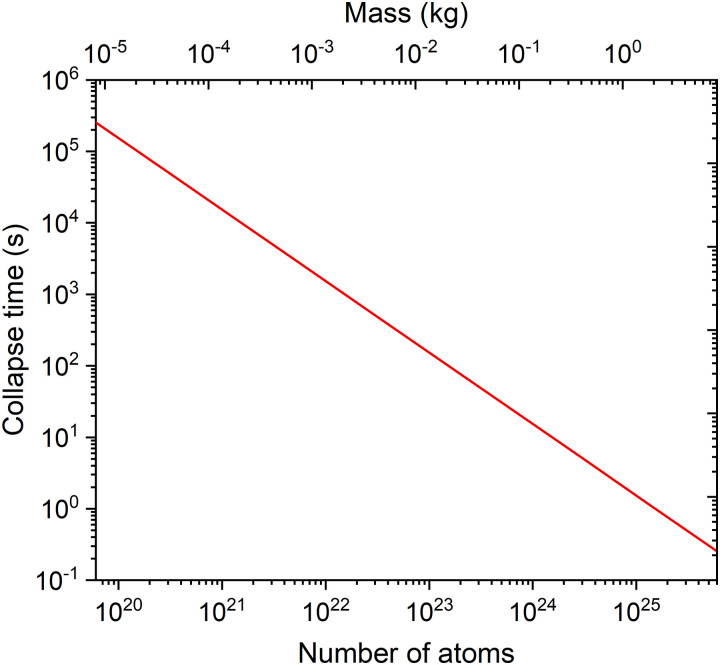
Diósi–Penrose collapse times computed with (9) as a function of the number of atoms and total mass for a system composed of Fe atoms in a quantum superposition of 10^14^ vacancies per mol. Note that the total mass corresponds to the entire crystal and not only of the atoms in the defects.

The result is shown in Fig. 7. According to the Diósi–Penrose model, the state superposition occurring in a 1 kg piece of iron would last about 1 s. This is a surprising result, meaning that if the Diósi–Penrose model is correct, quantum coherence could survive for a long time for macroscopic systems. However, observing it in a system like iron vacancy superposition at room temperature would still be challenging because decoherence (toward the crystal structure) would select the position basis and suppress any sign of quantum interference in a much shorter time scale.

**Fig. 8:**
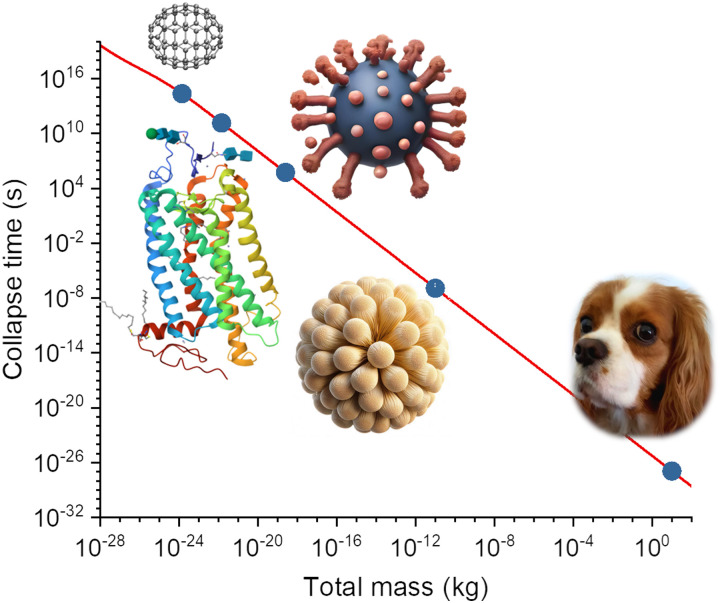
Minimum Diósi–Penrose collapse time computed with (8) for an isolated, homogeneous system composed of carbon atoms. The collapse times for equivalent masses of a fullerene (840 Da), a rhodopsin protein (85 kDa), an adenovirus (150 MDa), a pollen grain (10^−11^ kg), and a small dog (10 kg) are indicated, too.

When the superposition displacement increases, the gravitational self-energy saturates corresponding to the minimum collapse time, *τ*_C,min_. This quantity is plotted in Fig. 8 for a homogeneous carbon atom distribution, which, although artificial, can be taken as representative of the mean mass and width in organic matter. These values were obtained with (8) in the saturation regime (Δ*R* → ∞). δ*r̄* is computed with (C.10) for a number density of 7.5 × 10^28^ m^−3^, typical for coal. As expected, the collapse time is tiny for a macroscopic system, of the order of 10^−27^ s for a 1 kg mass. It increases to 10^−7^ s for 10^−11^ kg, the mass of a pollen grain. However, it is somewhat surprising that it is highly long for isolated mesoscopic systems like a virus (10^6^ s) or a protein (10^11^ s).

### 3.2 Proposed experimental setup

Suppose we want to observe the wave function collapse. In that case, we should guarantee (1) that it occurs within reasonable times (neither too long nor too short from the experimental perspective) and (2) that decoherence does not suppress the quantum interference before collapse takes place. We can control the first condition *via* the number of atoms, tuning the collapse time as we wish. The second condition, however, requires much more strict control of the environmental parameters. First, the entire system (as opposed to a subsystem) should be in the quantum superposition to avoid coherence delocalization over the environment. Second, for the same reason, the experiment should be in an extreme vacuum, possibly better than what we can achieve in conventional labs. Third, the system must be as close to absolute zero as possible to avoid decoherence due to the scattering of thermal photons and blackbody radiation.^18^

If these pressure and temperature conditions could be attained (in outer space, for instance^6^), we could consider a matter-wave scattering experiment in a Talbot–Lau interferometer like the one we discussed above in the case of C_70_. It would be set up such that the collapse time and the travel time between the last grating and the Tabolt point are similar. Then, one could use the mass to tune the ratio *τ*_C_/*t*_travel_ between these times. If the ratio is above one (*τ*_C_ > *t*_travel_), wave fringes should be observed at the Talbot point; if it is below one, the system would collapse before reaching *L*, and a Moiré pattern (expected for particles crossing the grating) would be observed.

**Fig. 9:**
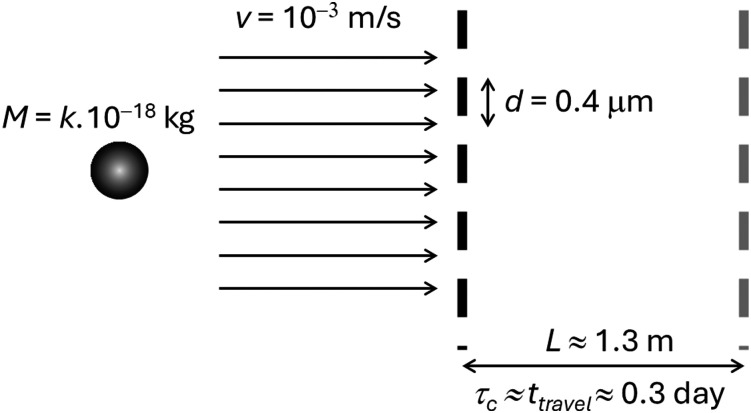
Parameters for a Talbot–Lau interferometer to verify the Diósi–Penrose collapse model. Only one of the gratings (in black) is illustrated. The parameters are set up to have similar travel (*t*_travel_) and collapse (*τ*_c_) times. Repeating the measurements with different masses (parameter *k*) would allow tuning the ratio between these times and observing either Talbot fringes (if *t*_travel_ < *τ*_c_) or Moir*é* patterns (*t*_travel_ > *τ*_c_).

Nevertheless, balancing the precise measurement conditions for such an experiment would still be a challenge. It could be achieved, for instance, by diffracting small, charged crystals of a dense material like osmium. If each crystal has a mass *M* = 5.6 × 10^−18^ kg, its radius is about 0.04 μm and could be diffracted at a grating with a *d* = 0.4 μm period (Fig. 9). It would have a Diósi–Penrose collapse time of just about 1334 s using (8) with *α* = 25.2 *a*_0_^−2^ (fitting in Table 1 with *R*_at_ = 2.44 Å given in ref. 47). If it is prepared at velocities as low as *v* = 10^−3^ m s^−1^, the Talbot length (*L* = *Mvd*^2^/*h*, where *h* is the Planck's constant) would be about *L* ≈ 1.3 m. With these parameters, the wave/particle would travel for about one-third of a day before reaching the first *L* point, which is (on purpose) near the collapse time.

To give an idea of how far we are from such a setup, some of the matter-wave diffraction studies with the biggest masses were made with molecules with about 10^−22^ kg (2000 atoms) and Talbot length under 2 m.^36^ In turn, quantum interference with wave packets separated by 0.5 m on a scale of 1 s was reported for ultracold atoms.^54^ Other proposed setups to verify the Diósi–Penrose model experimentally are discussed in ref. 6, 15, 30, 33 and 55–59.

### 3.3 Critical appraisal of the Diósi–Penrose collapse

The Diósi–Penrose model has been under intense theoretical^14,60^ and experimental^15,58^ scrutiny since it was proposed. In this section, we will outline a few points related to the atomistic approach to the model that needs to be clarified by further research.

#### 3.3.1 Mass distribution width

The width of the mass distribution has been under discussion since Diósi's initial proposal.^29,61^ In ref. 10, for instance, Diósi equaled it to 10^−5^ Å, a typical nuclear size. Penrose proposed that stationary-state nuclear wave functions, which are about 10^−2^ to 10^−1^ Å, provided a natural basis for mass distributions as long as their spreading in nonbonding systems was limited by a Newtonian gravitational potential.^9^ We agree that the nuclear wave function describes the mass distribution and, for molecules, in particular, it is automatically bound by the Born–Oppenheimer potential. This is indeed the approach we employed in all simulations of this paper, with the parameters of Table 1.

Nevertheless, non-interferometric experiments reported in ref. 15 (see also discussion in ref. 32), based on radiation emission of charged particles during the collapse, go in a different direction: they claim that the width of the mass distribution should be at least one order of magnitude broader than the nuclear wave function. For instance, for germanium atoms, the material they measured, they claimed that the mass distribution width must be bigger than *R*_0_ = 0.54 Å (where, in their model, *R*_0_ is the radius of a rigid sphere representing the nucleus). In contrast, using the fitting proposed in Table 1 (with *R*_at_ = 2.34 Å as given in ref. 47), the Gaussian standard deviation would be *R*_*N*_ = 0.07 Å. Comparing the sphere and the Gaussian radius is somewhat arbitrary. Still, if we overlook it, it is clear that *R*_*N*_ is smaller than the lower bound width *R*_0_.

The gravitational self-energy heuristically derived from Penrose's incompatibility measure in (2) has a free parameter *γ*. Here, we assumed without discussing that it is valued 1/(8π), following Howl, Penrose, and Fuentes.^16^ However, if this parameter is incorporated into the mass distribution *μ* (see eqn (3)), it would immediately imply that the Gaussian standard deviation *R*_*N*_ should be multiplied by a factor *γ*^−1/3^ to ensure that the integral over the mass distribution returns the nuclear mass, as expressed in (B.2). For the *γ* = 1/(8π), for example, all *R*_*N*_ values in Table 1 would be multiplied by 2.9. Any *γ* value below 0.002 would suffice to bring the Gaussian distribution above the lower bound set by Donadi *et al.*^15^

At this point, however, in view of all uncertainties associated with the gravitational self-energy and radiation rate estimates, we believe that it is still too early to rule out the nuclear wave function as the measure of mass distribution width, especially considering that there is no obvious replacement for it. Thus, it may be more fruitful to investigate the hypothesis that collapse in charged systems should be accompanied by radiation emission and invest in interferometric experiments to cross-check results.

#### 3.3.2 Preferred basis

In quantum mechanics, the wave function can be expanded on arbitrarily different bases. Still, we finally observe only a few bases, such as position. One of the biggest successes of the decoherence research program has been establishing that the continuous monitoring of the system by an environment selects a preferred system basis, the pointer states.^62,63^ These are the states with the least entanglement with the environment and that, therefore, survive to the decoherence.

Indifferent to this decoherence einselection, the Diósi–Penrose collapse necessarily implies that position should be the preferred basis. However, how should the collapse be in systems where the superposition is not expressed on a position basis? Consider, for example, superconducting quantum interference devices (SQUIDS),^64^ which show superpositions of clockwise and counter-clockwise flux in the device loop. In such a case, the gravitational self-energy is null, and collapse, according to the Diósi–Penrose model, would never occur.

It may be that the isolated description of the device is not enough to induce the collapse, and its correlations with the environment lead to position distortions that are ultimately responsible for it. This is the Bell conjecture,^65^ according to which “the only observations we must consider are position observations, if only the positions of instrument pointer.”

We commonly see such a type of indirect correlation in the nonadiabatic dynamics of molecules. During internal conversion, coherent superposition of different electronic states is formed. This superposition is not on a position basis but on the energy eigenstates. However, immediately after it is formed, the fractions of the nuclear wave packet on each electronic state follow different paths on the potential energy surfaces till their overlap is null, causing electronic decoherence^19^ and still selecting the energy eigenstates as pointer states. Thus, although the system's superposition is initially in the electronic energy eigenstates (not particularly sensitive to gravitationally induced collapse), the nuclear wave function is driven to a superposition of different positions, which in turn has a non-null gravitational self-energy and may collapse.

#### 3.3.3 Self-energy saturation

Another potential problem with the Diósi–Penrose model is the dependency of the gravitational self-energy on the superposition displacement Δ*R*. As expected, the gravitational self-energy increases when this displacement augments. However, it saturates to a maximum value, as shown in Fig. 5. For a homogeneous medium of identical nuclei, eqn (8) tells that this maximum value is12
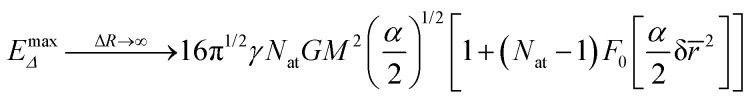
Thus, after a certain displacement, no matter how far the system in states A and B are pulled apart, the collapse time will always be about *τ*^min^_C_ = *ħ*/*E*^max^_*Δ*_, not depending on Δ*R*.

For comparison, decoherence has an entirely different dependency on the superposition displacement. The decoherence time over superposition displacement Δ*R* is proportional to Δ*R*^−2^, ensuring that decoherence becomes faster for more extensive separations.^48^

#### 3.3.4 Extensivity

The gravitational self-energy increases with the system's size, yielding extremely tiny collapse times if all atoms are displaced in the coherent state, as we can see in the examples of Fig. 8. However, in realistic situations, the quantum superposition may be restricted to small subregions of the system, with most of the molecular environment undisturbed. In such cases, the collapse time would be long, even for macroscopic objects. We saw such an example when we discussed the superposition of vacancies in iron crystals, where the effective size factor in (9) was harshly reduced to *N*_S_*M*^2^ (*N*_S_ ≪ *N*_at_). That was also the case of sodium ion superpositions in a neuron (see Table 2), where only about a million atoms matter for the coherent state.

Another example of this extensivity problem may occur in the photoisomerization of retinal chromophores in proteins. Despite the significant conformational changes that the chromophore experiences when moving from the *cis* to *trans* conformations, the geometric distortions in the protein and water environments are limited. That is so to the point that in advanced atomistic simulations, only the sidechain atoms of the residues forming the cavity around the retinal and a few water molecules are geometrically relaxed.^66^ Most and all remaining protein is unchanged. It could be that this initial photoisomerization process, with limited geometric displacements, does not reflect later, more extensive geometric distortions^67^ that could enhance the gravitational self-energy. However, it could equally be that these later distortions are still restricted to the immediate environment, implying a long collapse time.

We could always argue that when the quantum state of the systems entangles with the environment, the measuring apparatus, and the observer through the von Neumann chain,^17^ the gravitational self-energy increases, shortening the collapse times. A 1-cm aluminum instrument pointer of 1 g in a two-state superposition 1 cm apart would instantaneously collapse within 6 × 10^−21^ s (in a direct application of (8), with *α* = 25.2 *a*_0_^−2^ and δ*r̄* given by (C.10) with *n* = 6 × 10^28^ m^−3^). However, such reasoning defeats the purpose of the collapse theories, which is to attribute a naturally occurring collapse time independent of any laboratory measurement.

## 4. Conclusions

Wave function collapse is postulated in standard quantum mechanics to explain how the only possible result of the measurement of a physical quantity is one of the eigenvalues of the corresponding observable.^1^ However, standard quantum mechanics does not explain how this nonunitary process occurs; it only states its probability. In the frame of objective collapse theories, which modify the Schrödinger unitary propagation to account for collapse, Diósi^29^ and, independently, Penrose^8^ proposed that gravity induces collapse within a time inversely proportional to the gravitational self-energy of the quantum superposition.

Here, we developed models to compute the Diósi–Penrose collapse time in general atomistic systems (eqn (6)) and homogeneous media (eqn (8) and (9)). We applied these models for systems ranging from a small isolated molecule, for which the collapse time is of the order of a billion years, to mesoscopic crystals, whose collapse could occur within days, and macroscopic systems, collapsing within a rontosecond (10^−27^ s). We also discuss collapse in the context of decoherence, which is another essential element in the route from quantum to classical, and propose an interferometric experiment to verify the Diósi–Penrose hypothesis.

We critically appraised the Diósi–Penrose collapse model, discussing diverse points that need to be clarified by further research. They include the width of the mass distribution, whose main proposal is at odds with non-interferometric experimental results; the selection of preferred basis, whose status is not as straightforward as in the decoherence program; the gravitational self-energy saturation, which leads to a nonintuitive result where the collapse time does not depend on the superposition displacement; and the extensivity problem, which seems to imply that macroscopic system may have too long collapse times.

It is still early to state if the Diósi–Penrose model can solve the quantum measurement problem. However, the several drawbacks pointed out by previous research and by the present work may indicate that some reformulation of the theory is needed to retain gravity-induced collapse theories as viable contenders among the many proposed solutions to the problem.

This paper deals with a subject that dates to the beginning of quantum mechanics, has no formed consensus, splits physicists and philosophers, and is still rapidly evolving. Thus, it is impossible to credit all theories and authors. For instance, although we focused on the Diósi–Penrose model, it is not the only objective collapse theory^4^ and not even the only proposal to modify quantum mechanics to incorporate gravity.^21,22,68^

No field in physics invokes as many passionate philosophical debates as quantum mechanics. Interpretation of quantum mechanics became so problematic that even outstanding scientists seemingly gave up hope that we may reach a scientific consensus. They think that the interpretation of quantum mechanics is fated to be a matter of choice.^69^ We do not share this opinion. As scientists, we must seek a realist (pretty much as Einstein, Podolsky, and Rosen famously defined^70^) and experimentally verifiable interpretation of the quantum world, even if this interpretation costs us some foundational stone like locality^71,72^ or means that we live in a universe where statistic independence is a conceptual impossibility.^73,74^

Zeh, who developed the early concepts of what would later be known as decoherence, considered the collapse a troublesome superfluous hypothesis: “Any deviation from the global Schrödinger equation must, in principle, lead to observable effects, and it should be recalled that none have ever been discovered.”^75^ However, proposing ways and attempting to verify such effects are worth it. It may come the day we would have to bow to a realist but unverifiable hypothesis based on some relative state interpretation^20,76^ or admit that the wave function is a subjective construct.^27,28^ But we are not quite there yet. Experiments such as the cosmic Bell tests,^77^ the gradual formation of eigenstates,^78^ or those reconstructing wave function phases out of measurements^79^ show we still have much to learn. The Diósi–Penrose model also tells us that we have room for realist, testable theories of measurement. We hope our atomistic modes of the Diósi–Penrose will help in this endeavor.

## Data availability

All data supporting this article can be directly recomputed with the equations given in Section 2.

## Conflicts of interest

There are no conflicts to declare.

## Figures and Tables

**Fig. 10:**
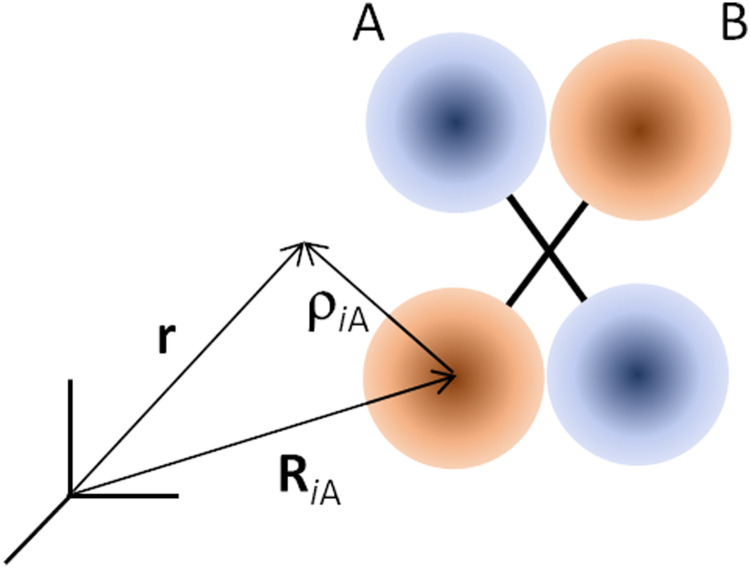
Scheme of the A–B superposition of a two-nuclei system.

## Appendices

### Appendices

#### A Penrose's incompatibility measure

We can develop *E*_*Δ*_ expressed in (2) in terms of the Newtonian gravitational potentials *ϕ*_A_ and *ϕ*_B_ of states A and B to obtain

A.1

where we used **a**_S_ = −∇*ϕ*_S_.

The gravitational potential *ϕ*_S_ at point **r** due to the mass density *μ*_S_ at point **r**′ are related either by the Poisson's equation

A.2

or by the Green's function

A.3

Using these relations, *E*_*Δ*_ becomes

A.4

#### B Derivation of the gravitational self-energy

##### B.1 Gaussian particles distribution

To drive the gravitational self-energy, we neglect the electron masses and exclusively focus on the nuclear distribution. We assume that each nucleus *i* in state A has a Gaussian mass distribution

B.1

Centered in **R**_*i*A_ with width *α*_*i*_ and decreasing with a radial distance ***ρ***_*i*A_ from this point. This mass distribution should integrate into the full nuclear mass *M*_*i*_, such that

B.2

The full mass density of state A at point **r** is a sum over the mass distributions of all *N*_at_ nuclei

B.3

where ***ρ***_*i*A_(**r**) is

B.4 ***ρ***_*i*A_(**r**) = **r** − **R**_*i*A_

as illustrated in Fig. 10.

Following the same steps, the mass distribution of state B is equivalently written as

B.5

where *β*_*i*_ is the Gaussian width of nucleus *i* in state B.

Now, we turn our attention to the integral in (3). It can be expanded as

B.6

The last term is composed of integrals of the product of mass distributions. Let us take, for example, the integral involving the product *μ*_A_(**r**′)*μ*_B_(**r**). Replacing the mass distributions (B.3) and (B.5) into that integral renders

B.7

This type of integrals is analogous to 1s two-electron integrals^80^ and can be solved analytically, as discussed next.

##### B.2 Analytical Gaussian integrals

Consider the Gaussian functions of the type

B.8

The four-center integral is (see eqn (A.41) of ref. 80)

B.9

where

B.10

and

B.11

We can recast the integral (B.7) into such a format by rewriting the Gaussian mass distribution (B.1) in the format of the Gaussian functions in (B.8). For instance

B.12

Therefore,

B.13

According to (B.9), this integral is

B.14

Thus,

B.15

The other integrals we will need to solve (B.6) are

B.16

B.17

B.18

##### B.3 Gravitational self-energy

Using (B.15), integral (B.7) becomes

B.19

We follow similar steps using eqn (B.16)–(B.18) for the other integrals in (B.6)

B.20

B.21

B.22

Finally, the total integral (B.6) is

B.23

In (B.23), the factor 2 in the last term in the right hand comes from recognizing that

B.24 (BB|AA) = (AA|BB)

Returning to (3), the gravitational self-energy for an ensemble of Gaussian particles is

B.25

##### B.4 Sanity check: the single-particle limit

We can check our result (B.25) by comparing it with the single-particle result that Howl, Penrose, and Fuentes derived for Bosen–Einstein condensates in the Gaussian approximation using a different approach.^16^ If the system has a single particle, (B.25) simplifies to

B.26

Considering that the Gaussian width is the same in the two states, we get from (B.26)

B.27

with the distance between the two states given as *b* ≡ |**R**_A_ − **R**_B_|. We can also write *α* = 1/(*kR*^2^), where *R* is the radius of the Gaussian and recast the equation in terms of *λ* ≡ *b*/(2*k*^1/2^*R*)

B.28

In these equations, *k* is an open parameter. In particular, when *k* = 2, *R* is the Gaussian standard deviation.

In ref. 16, *γ* is taken as 1/(8π). We do the same here to obtain

B.29

where *R*′ = *k*^1/2^*R*, which is the same result obtained by Howl, Penrose, and Fuentes.^16^

#### C *E*_*Δ*_ in a homogeneous, isotropic, uniform medium

Supposing that both states in the superposition share the same Gaussian width and all nuclei are identical, (B.25) becomes

C.1

Now, we separate diagonal and nondiagonal terms to write

C.2

where

C.3

and

C.4

For the diagonal terms, we assume the same scalar displacement Δ*R* between equivalent atoms in states A and B, which implies

C.5

To estimate *f*_*ij*_, we assume

C.6 |**R**_*i*A_ − **R**_*j*A_| = |**R**_*i*B_ − **R**_*j*B_| ≈ δ*r̄*

where δ*r̄* is the mean distance between any pair of atoms in the system. We also assume that the direction of δ*r̄* is approximately perpendicular to the direction of Δ*R*, which implies

C.7

With these approximations, *f*_*ij*_ is

C.8

If the particular case of *N*_at_ spherically distributed identical atoms with number density *n* (atom/volume), these atoms fill a sphere of radius

C.9

Then, the mean distance between any two points *i* an *j* within a sphere of radius *ρ* is

C.10

Now, we use (C.5) and (C.8) to evaluate the sums in (C.2):

C.11

and

C.12

Thus, the self-energy in this approximation is

C.13

If δ*r̄* ≫ Δ*R*, it simplifies to

C.14
